# Identification of SMAD3 as a Novel Mediator of Inflammation in Human Myometrium *In Vitro*

**DOI:** 10.1155/2018/3140420

**Published:** 2018-09-27

**Authors:** Martha Lappas

**Affiliations:** ^1^Obstetrics, Nutrition, and Endocrinology Group, Department of Obstetrics and Gynaecology, University of Melbourne, Victoria, Australia; ^2^Mercy Perinatal Research Centre, Mercy Hospital for Women, Heidelberg, Victoria, Australia

## Abstract

Preterm birth remains the primary cause of early neonatal death and is a major determinant for long-term health consequences. Aberrant intrauterine inflammation and infection are known to augment the synthesis of proinflammatory cytokines and induce uterine contractions, which can subsequently lead to preterm birth. The transforming growth factor-*β* (TGF-*β*) superfamily members regulate numerous cellular processes through the activation of intracellular mediators known as mothers against decapentaplegic homolog (SMADs). Studies in nongestational tissues have shown that SMAD3 plays a role in immune regulation and inflammation; however, its role in human labour remains unknown. Thus, the present study aimed at (i) characterising the expression of SMAD3 in the human myometrium; (ii) determining the effect of bacterial and viral products and proinflammatory cytokines on SMAD3 transcriptional activity in primary human myometrial cells; and (iii) investigating the effect of SMAD3 siRNA knockdown on the production of prolabour mediators in primary human myometrial cells. Phosphorylated (i.e., active) SMAD3 protein expression was lower in the myometrium after spontaneous term labour compared to the myometrium from nonlabouring women. Using a luciferase assay, the proinflammatory cytokines IL-1*β* and TNF, and viral analogue polyinosinic : polycytidylic acid (poly(I : C)) significantly reduced SMAD3 transcriptional activity in human primary myometrial cells. Loss-of-function studies found that SMAD3 knockdown in myometrial cells significantly increased IL-1*β*- and poly(I : C)-induced proinflammatory cytokines (IL-1A, IL-6), chemokines (IL-8, MCP-1), the adhesion molecule ICAM-1, COX-2 mRNA expression, and subsequent PGF_2*α*_ release. In conclusion, SMAD3 deficiency is associated with increased production of proinflammatory and prolabour mediators in the human myometrium.

## 1. Introduction

The most common cause of early neonatal death is preterm birth. It is responsible for approximately 1 million neonatal deaths globally each year [1]. Preterm infants have greatly increased rates of long-term disabilities including cerebral palsy, intellectual handicap, and chronic lung disease [2]. Such complications lead to long-term morbidity through childhood and extend into adult life, casting an enormous financial burden on the health system [3].

Aberrant intrauterine inflammation and/or pathological processes (e.g., maternal viral or bacterial infection) are frequently associated with preterm birth by initiating uterine contractions [4, 5]. Indeed, *in vivo* animal studies have shown that administration of the proinflammatory cytokine IL-1*β* or the synthetic analog of viral dsRNA polyinosinic : polycytidylic acid (poly(I : C)) can induce preterm birth [6, 7]. The presence of intrauterine infection and/or inflammation results in the activation of the maternal immune response and the influx of leukocytes into the myometrium and cervix and increased production of proinflammatory cytokines such as IL-1*β* and TNF within these tissues [8, 9]. In addition to further inducing cytokines, IL-1*β* and TNF can also induce chemokine production (such as IL-8 and MCP-1) [10] or increase the production of phospholipid-derived mediators (such as prostaglandins) which are known to play an important role in myometrial contractions and cervical remodelling [5, 11–14].

Mothers against decapentaplegic homolog (SMADs) play a critical role in regulating the expression of genes associated with inflammatory activation [15–19]. There are three classes of SMADs: (i) regulatory SMADs (R-SMADs) which include SMAD1, SMAD2, SMAD3, SMAD5, and SMAD8/9; (ii) common SMADs (Co-SMADs) which include only SMAD4; and (iii) inhibitory SMADs (I-SMADs) which include SMAD6 and SMAD7, which block the activation of receptor-regulated and common-mediator SMADs. In the canonical pathway, transforming growth factor- (TGF-) *β* binds to TGF-*β* receptors to stimulate the phosphorylation of receptor-regulated SMAD proteins (e.g., phosphorylation of SMAD3 at two C-terminal Ser residues, Ser-423 and Ser-425), which in turn form complexes with SMAD4 that accumulate in the nucleus [20]. In the nucleus, SMAD proteins can bind directly to their cognate DNA-binding sites to activate or inhibit transcription, regulating gene expression of target genes associated with inflammatory activation. In the nucleus, SMADs can interact with an increasing number of transcription factors, transcriptional coactivators, or transcriptional corepressors.

SMAD3 serves as a target for other mediators independent of the canonical TGF-*β* pathway. Phosphorylation by p38 mitogen-activated protein kinase (MAPK; a key regulator of *proinflammatory* cytokines), cotranscription factors, and I-SMADs are also known to regulate SMAD3-driven gene transcription [21]. SMAD3 is an anti-inflammatory transcription factor. Mice lacking functional SMAD3 respond to bacterial lipopolysaccharide (LPS) with greater mortality than their wild-type littermates [22]: there is enhanced vascular inflammation induced by LPS in SMAD3 knockout mice [18]; SMAD3 null mast cells showed enhanced production of proinflammatory cytokines upon LPS stimulation [23]; and inhibition of SMAD2/3 increases LPS-mediated inflammation in macrophages [24]. Conversely, the overexpression of SMAD3 inhibits LPS-induced inflammation [25]. In addition, inflammatory insults can also impair SMAD3 expression. For example, brains affected by Alzheimer disease are associated with decreased levels of SMAD3 [26], while SMAD3 phosphorylation (i.e., activity) is reduced in proximal tubular cells stimulated with IL-1*β* [27] and in cardiac fibroblasts treated with LPS [28].

There is limited data on SMADs and pregnancy. Animal studies have that shown decidualisation of endometrial stroma is compromised with SMAD3 deficiency [29]. Furthermore, TGF-*β* is overexpressed in preeclamptic placenta [30]. Failure to downregulate overexpressed TGF-*β* during early gestation may cause shallow trophoblast invasion and predisposes the pregnancy to preeclampsia [30]. In contrast, TGF-*β* treatment can improve adverse pregnancy outcomes caused by *T. gondii* infection by upregulating Treg cell differentiation and function via upregulating SMAD3 expression [31].

We have previously established that SMAD7 expression is increased in labouring myometrium and by mediators of labour [32]. Furthermore, we found that SMAD7 is involved in the regulation of prolabour mediators induced by inflammatory insults [32]. There is, however, no data on the role of SMAD3 and human labour. Thus, we hypothesised that (i) human labour and delivery would be associated with decreased SMAD3 expression in the myometrium; (ii) proinflammatory stimuli would decrease SMAD3 transcriptional activity; and (iii) loss-of-functioning SMAD3 using siRNA would be associated with increased expression and secretion of labour mediators in the presence of inflammatory insults. Thus, the aims of this study were to investigate the effect of (i) human spontaneous term labour on SMAD3 expression in the human myometrium; (ii) bacterial and viral products and proinflammatory cytokines on SMAD3 transcriptional activity in primary cells isolated from the myometrium; and (iii) SMAD3 siRNA on proinflammatory and prolabour mediators in primary myometrium cells.

## 2. Materials and Methods

### 2.1. Tissue Collection

Human myometrium was obtained (with institutional Research and Ethics Committee approval) from the upper margin of the lower uterine segment incision during Caesarean section. Myometrium was processed as previously described [33–36]. Tissues were immediately snap frozen in liquid nitrogen and stored at −80°C for expression studies, or used immediately for cell culture experiments. Exclusion criteria is as previously described [32, 34, 35] and included diabetes, asthma, polycystic ovary syndrome, preeclampsia, multiple pregnancies, obesity, and fetuses with chromosomal abnormalities.

### 2.2. Expression Studies

To characterise labour-associated changes in the SMAD3 expression, myometrium was obtained from pregnant women undergoing elective Caesarean section either in the absence of labour (*n* = 8 patients) or during active labour (*n* = 8 patients; average length of labour was 10 h ± 6 h 40 min) as previously described [32, 34, 35]. Labour was defined as the presence of regular uterine contractions (every 3–4  min) resulting in cervical effacement and dilation; none of these patients received any medications to augment or induce labour. Indications for Caesarean section in the absence of labour were breech presentation and/or previous Caesarean section. Indications for Caesarean section in the labouring samples were for placenta praevia, fetal distress, and delayed or failure to progress. Tissue samples were snap frozen in liquid nitrogen and immediately stored at −80°C for analysis by Western blot as detailed below.

### 2.3. SMAD3 siRNA Transfection in Primary Human Myometrial Cells

Primary human myometrial cells were used to determine the effect of SMAD3 knockdown on the expression of prolabour mediators. Myometrial cells (*n* = 5 patients) were isolated and cultured as previously described [33–36]. Transfection was performed as we have previously described [34–37] using SMAD3 siRNA (50 nM siSMAD3; Ambion (Thermo Fisher Scientific; Scoresby, Vic, Australia)) and negative control siRNA (50 nM siCONT; Ambion (Thermo Fisher Scientific; Scoresby, Vic, Australia)). Following 48 h of transfection, cells were treated with 1 ng/ml IL-1*β* or 5 *μ*g/ml poly(I : C) for 24 h; cells and conditioned media were collected separately and stored at −80°C until analysed as detailed below. Cell viability was assessed by the 3-(4,5-dimethyl-2-thiazolyl)-2,5-diphenyl-2H-tetrazolium bromide (MTT) proliferation assay as we have previously described [38]. The experiments were performed from myometrium obtained from five patients.

### 2.4. SMAD3 Luciferase Assay

A luciferase assay was utilised to determine the effect of inflammatory insults on SMAD3 activity, as previously described [33, 36] with minor modifications. Primary myometrial cells (*n* = 5 patients) were transfected with 300 ng SMAD3 reporter construct (Qiagen; Chadstone Centre, Vic, Australia) using FuGENE HD transfection reagent (Promega; Alexandria NSW, Australia) for 48 h, then treated with or without 1 ng/ml IL-1*β*, 10 ng/ml TNF, 5 *μ*g/ml poly(I : C), 250 ng/ml fsl-1, 1 *μ*g/ml flagellin, or 10 ng/ml TGF-*β*1 for an additional 20 h. Luminescence activity was measured using a Luciferase Reporter Assay Kit (Life Research; Scoresby, Vic, Australia) and Renilla Luciferase Flash Assay kit (Thermo Fisher Scientific; Scoresby, Vic, Australia) as instructed and previously described [33].

### 2.5. RNA Extraction and qRT-PCR

RNA extractions, cDNA synthesis, and qRT-PCR were performed as previously described [32, 35, 36, 39]. The RT-PCR was performed using the CFX384 Real-Time PCR detection system (Bio-Rad Laboratories; Gladesville, NSW, Australia) using 100 nM of predesigned and validated QuantiTect primers (primer sequences not available) (Qiagen; Chadstone Centre, Vic, Australia). Average gene *C*_*t*_ values were normalised against two housekeeping genes (*β*2-Microglobulin (B2M) and succinate dehydrogenase complex subunit A (SDHA)). Of note, there was no effect of experimental treatment on B2M or SDHA mRNA expression. Fold differences were determined using the comparative *C*_*t*_ method.

### 2.6. Western Blotting

Western blotting was performed as previously described [34, 35, 40]. Blots were incubated in 1 *μ*g/ml goat polyclonal antiphosphorylated SMAD3 (p-SMAD3; Santa Cruz Biotechnology; Santa Cruz, CA, USA) or 1 *μ*g/ml mouse monoclonal anti-SMAD3 (Santa Cruz Biotechnology; Santa Cruz, CA, USA) prepared in blocking buffer (5% skim milk in TBS (50 mM Tris-Cl, pH 7.5, 150 mM NaCl) with 0.05% Tween-20) for 16 h at 4°C. Semiquantitative analysis of the relative density of the bands in Western blots was performed using Quantity One 4.2.1 image analysis software (Bio-Rad Laboratories, Hercules, CA, USA).

### 2.7. Enzyme Immunoassays

Assessment of cytokine and chemokine release of IL-6 and IL-8 was performed using the CytoSet™ sandwich ELISA according to the manufacturer's instructions (Life Technologies; Mulgrave, Vic, Australia). The release of MCP-1, sICAM-1, and sVCAM-1 was performed by sandwich ELISA from R&D Systems (Minneapolis, MN, USA) according to the manufacturer's instructions. The release of PGF_2*α*_ into the incubation medium was assayed using a commercially available competitive enzyme immunoassay kit according to the manufacturer's specifications (Cayman Chemical Company; Ann Arbor, MI, USA). The interassay and intraassay coefficients of variation for all assays were less than 10%.

### 2.8. Statistical Analysis

All statistical analyses were undertaken using GraphPad Prism (GraphPad Software, La Jolla, CA, USA). For two sample comparisons, either a paired or unpaired Student's *t*-test was used to assess statistical significance between normally distributed data; otherwise, the nonparametric Mann–Whitney *U* (unpaired) or the Wilcoxon (matched pairs) tests were used. For all other comparisons, the homogeneity of data was assessed by Bartlett's test, and when significant, data were logarithmically transformed before analysis by a repeated measures one-way ANOVA (with LSD post hoc testing to discriminate among the means). Statistical significance was ascribed to a *P* value ≤ 0.05. Data is expressed as mean ± SEM.

## 3. Results

### 3.1. Expression of SMAD3 in Myometrium

**Figure 1:**
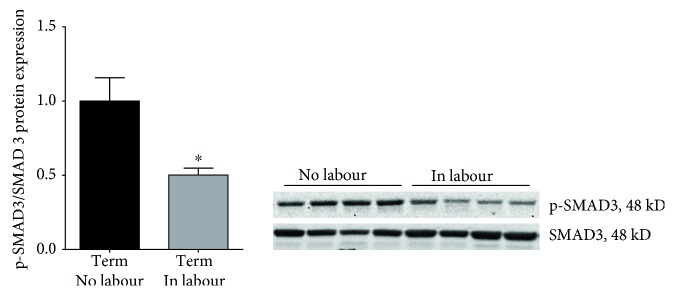
Expression of SMAD3 in the myometrium. Human myometrium was obtained from nonlabouring (term no labour, *n* = 8 patients) and labouring women at term Caesarean section (term in labour, *n* = 8 patients). Phosphorylation of SMAD3 at serine 423/425 (p-SMAD3) was analysed by Western blotting. Phosphorylated SMAD3 protein expression was normalised to total SMAD3 protein expression and the fold change was calculated relative to the no labour group. Data is displayed as mean ± SEM. ^∗^ *P* ≤ 0.05 vs. term no labour (Mann–Whitney *U* test). A representative Western blot from 4 patients per group is also shown.

Human myometrium was obtained at term Caesarean section in the absence of labour (term, no labour) and at term Caesarean section during spontaneous labour onset (term, in labour). Serine phosphorylation of SMAD3 is required for the activation of SMAD3 [41]. Thus, the protein expression of total and phosphorylated Ser 423/425 SMAD3 (p-SMAD3) was assessed by Western blotting; data are expressed as p-SMAD3 expression relative to total SMAD3 levels. As presented in Figure 1, p-SMAD3 expression was significantly lower in the myometrium after spontaneous labour at term when compared to nonlabouring tissues.

**Figure 2:**
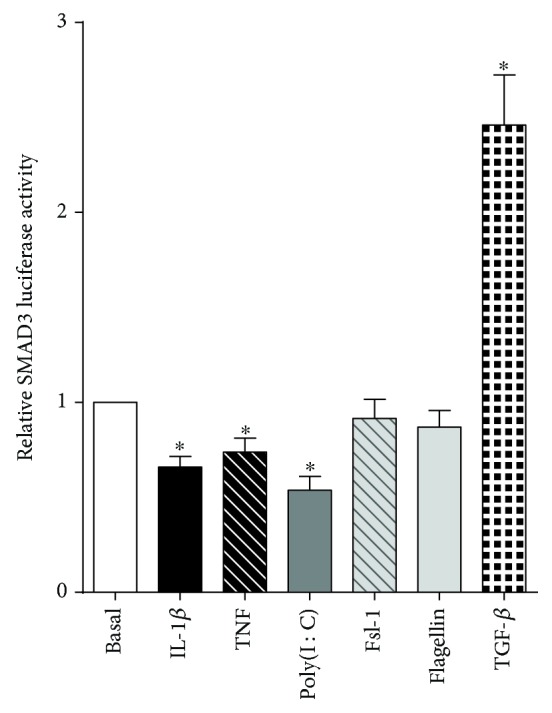
Effect of proinflammatory mediators on SMAD3 activity. Human primary myometrial cells, obtained from women at term in the absence of labour, were cotransfected with 300 ng SMAD3-luc reporter construct for 48 h, then treated for an additional 24 h with 1 ng/ml IL-1*β*, 10 ng/ml TNF, 5 *μ*g/ml poly(I : C), 250 ng/ml fsl-1, 1 *μ*g/ml flagellin, or 10 ng/ml TGF-*β* (*n* = 5 patients per treatment). Promoter activity is expressed as a ratio of luciferase activity of the SMAD3 reporter under basal conditions. Each bar represents the mean ± SEM. ^∗^ *P* ≤ 0.05 vs. basal (paired sample comparison).

A range of known mediators of spontaneous preterm birth were used to determine which could affect SMAD3 activity in the myometrium. We tested the proinflammatory cytokines IL-1*β* and TNF; a range of toll-like receptor (TLR) ligands including the viral dsRNA analogue poly(I : C) (TLR3 ligand); and the bacterial products fsl-1 (TLR2/6 ligand) and flagellin (TLR5 ligand). Treatment of myometrial cells with IL-1*β*, TNF, and poly(I : C) significantly decreased SMAD3 transcriptional activity (Figure 2). There was, however, no effect of fsl-1 or flagellin on SMAD3 activity. TGF-*β* was used as a positive control, and, as expected, there was a 2.5-fold increase in SMAD3 transcriptional activity.

### 3.2. Effect of SMAD3 siRNA Knockdown on Proinflammatory Cytokines and Chemokines

Given that IL-1*β* and poly(I : C) significantly decreased SMAD3 transcriptional activity in myometrial cells, functional siRNA studies were next undertaken to investigate the role of SMAD3 in the genesis of prolabour mediators induced by IL-1*β* or poly(I : C). The efficacy of siSMAD3 knockdown is demonstrated in Supplementary Figure S1. There was an 80% decrease in SMAD3 mRNA expression and a 70% decrease in SMAD3 protein expression. A MTT cell viability assay showed no difference in absorbance between cells transfected with siCONT or siSMAD3 (Supplementary Figure S1).

**Figure 3:**
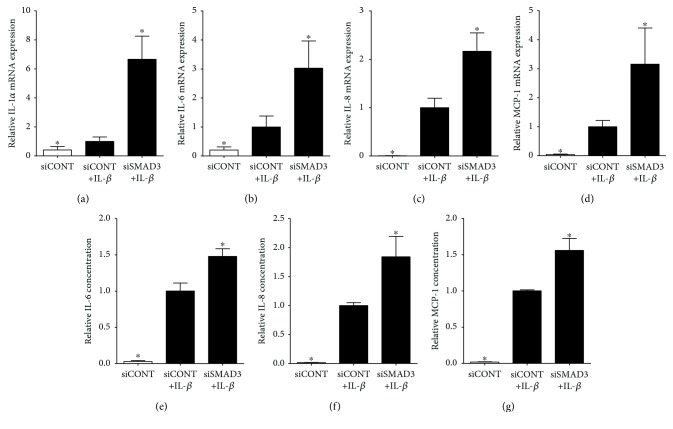
Effect of siSMAD3 on IL-1*β*-induced proinflammatory cytokines and chemokines. Human primary myometrial cells, obtained from women at term in the absence of labour, were transfected with or without 50 nM siSMAD3 or 50 nM siCONT for 48 h and then treated with 1 ng/ml IL-1*β* for an additional 24 h (*n* = 5 patients). (a–d) Expression of IL-1*α*, IL-6, IL-8, and MCP-1 mRNA was analysed by qRT-PCR. (e–g) IL-6, IL-8, and MCP-1 concentrations in the incubation medium were assayed by ELISA. For all data, the fold change was calculated relative to IL-1*β*-stimulated siCONT-transfected cells and displayed as mean ± SEM. ^∗^ *P* ≤ 0.05 vs. IL-1*β*-stimulated siCONT-transfected cells (one-way ANOVA).

**Figure 4:**
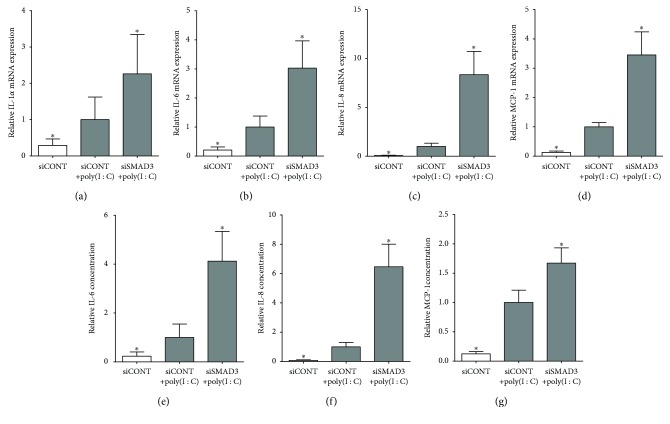
Effect of siSMAD3 on poly(I : C)-induced proinflammatory cytokines and chemokines. Human primary myometrial cells, obtained from women at term in the absence of labour, were transfected with or without 50 nM siSMAD3 or 50 nM siCONT for 48 h and then treated with 5 *μ*g/ml poly(I : C) for an additional 24 h (*n* = 5 patients). (a–d) Expression of IL-1*α*, IL-6, IL-8, and MCP-1 mRNA was analysed by qRT-PCR. (e–g) IL-6, IL-8, and MCP-1 concentrations in the incubation medium were assayed by ELISA. For all data, the fold change was calculated relative to poly(I : C)-stimulated siCONT-transfected cells and displayed as mean ± SEM. ^∗^ *P* ≤ 0.05 vs. poly(I : C)-stimulated siCONT-transfected cells (one-way ANOVA).

For all following studies, after siRNA transfection, cells were treated with or without IL-1*β* or poly(I : C) to induce the expression and secretion of prolabour mediators. As expected, in siCONT-transfected cells, treatment of myometrium cells with IL-1*β* (Figure 3) or poly(I : C) (Figure 4) significantly increased IL-1*α*, IL-6, IL-8, and MCP-1 mRNA expression and IL-6, IL-8, and MCP-1 secretion. The effect of transfection with siSMAD3 was a significant increase in IL-1*β*- (Figure 3) and poly(I : C)-induced (Figure 4) IL-1α, IL-6, IL-8, and MCP-1 mRNA expression and secretion of IL-6, IL-8, and MCP-1. As we have previously reported, IL-1*α* levels are not detectable in the incubation media from human primary myometrial cells [42] and were thus not assessed.

### 3.3. Effect of SMAD3 siRNA Knockdown on Adhesion Molecules

**Figure 5:**
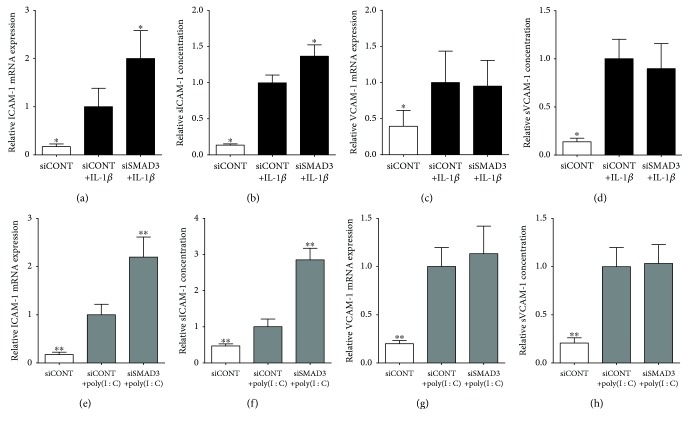
Effect of siSMAD3 on the expression and secretion of adhesion molecules. Human primary myometrial cells, obtained from women at term in the absence of labour, were transfected with or without 50 nM siSMAD3 or 50 nM siCONT for 48 h and then treated with (a–d) 1 ng/ml IL-1*β* or (e–h) 5 *μ*g/ml poly(I : C) for an additional 24 h (*n* = 5 patients). (a, c, e, g) Expression of ICAM-1 and VCAM-1 mRNA was analysed by qRT-PCR. (b, d, f, h) sICAM-1 and sVCAM-1 concentrations in the incubation media were assayed by ELISA. The fold change was calculated relative to IL-1*β*- or poly(I : C)-stimulated siCONT-transfected cells and data displayed as mean ± SEM. ^∗^ *P* ≤ 0.05 vs. IL-1*β*-stimulated siCONT-transfected cells (one-way ANOVA). ^∗∗^ *P* ≤ 0.05 vs. poly(I : C)-stimulated siCONT-transfected cells (one-way ANOVA).

We next sought to determine the effect of SMAD3 siRNA on the expression of adhesion molecules. As shown in Figure 5, in siCONT-transfected cells, IL-1*β* or poly(I : C) treatments significantly increased ICAM-1 and VCAM-1 mRNA expression and secretion. In siSMAD3-transfected cells, IL-1*β*- and poly(I : C)-induced ICAM-1 mRNA expression and secretion was significantly increased (Figures 5(a), 5(b), 5(e), and 5(f)). In contrast, there was no effect on VCAM-1 mRNA expression and secretion with siSMAD3 transfection (Figures 5(c), 5(d), 5(g), and 5(h)).

### 3.4. Effect of SMAD3 siRNA Knockdown on COX-2-Prostaglandin Pathway

**Figure 6:**
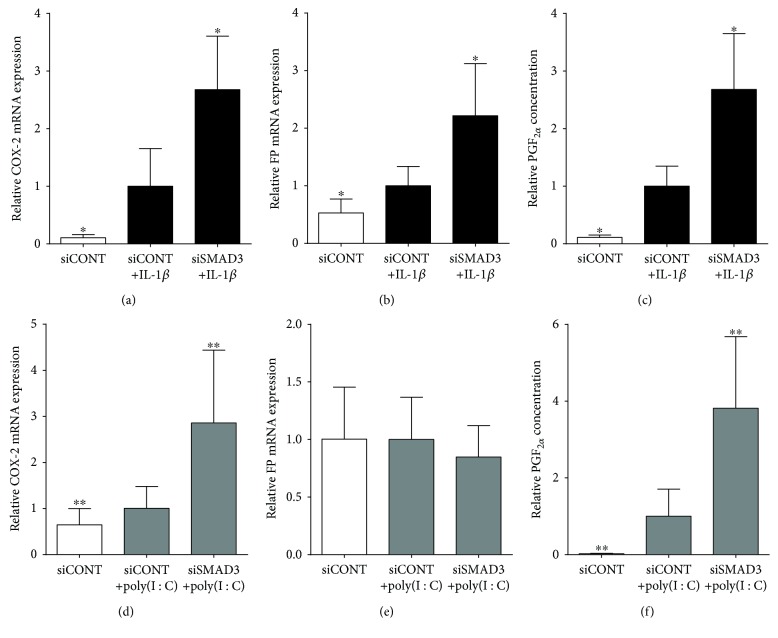
Effect of siSMAD3 on the COX-2-prostaglandin pathway. Human primary myometrial cells, obtained from women at term in the absence of labour, were transfected with or without 50 nM siSMAD3 or 50 nM siCONT for 48 h and then treated with (a–c) 1 ng/ml IL-1*β* or (d–f) 5 *μ*g/ml poly(I : C) for an additional 24 h (*n* = 5 patients). (a, c, d, e) Expression of COX-2 and FP mRNA was analysed by qRT-PCR. (c, f) PGF_2*α*_ concentration in the incubation medium was assayed by ELISA. For all data, the fold change was calculated relative to IL-1*β*- or poly(I : C)-stimulated siCONT-transfected cells and displayed as mean ± SEM. ^∗^ *P* ≤ 0.05 vs. IL-1*β*-stimulated siCONT-transfected cells (one-way ANOVA). ^∗∗^ *P* ≤ 0.05 vs. poly(I : C)-stimulated siCONT-transfected cells (one-way ANOVA).

The final aim of this study was to determine the effect of SMAD3 loss-of-function on the COX-2-prostaglandin pathway in the presence of IL-1*β* or poly(I : C). As expected, in siCONT-transfected cells, treatment with IL-1*β* significantly increased COX-2 and FP mRNA expression and PGF_2*α*_ release (Figures 6(a)–6(c)). This increase was significantly augmented in siSMAD3-transfected cells. In siCONT-transfected cells, treatment with poly(I : C) significantly increased COX-2 mRNA expression and PGF_2*α*_ release (Figures 6(d) and 6(f)). Transfection with siSMAD3 significantly increased this poly(I : C)-induced response. There was no effect of poly(I : C) with or without siSMAD3 on FP mRNA expression (Figure 6(e)).

## 4. Discussion

This study has shown, for the first time, the labour-associated changes in SMAD3 expression and its anti-inflammatory role in the human myometrium. The data presented in this study demonstrates that SMAD3 activity is reduced in the myometrium with term labour, as well as in response to proinflammatory cytokines (IL-1*β* and TNF) or TLR3 activation (poly(I : C)). Loss-of-function studies were also performed in primary myometrial cells to determine the effect of SMAD3 siRNA on the expression and release of proinflammatory and prolabour mediators. The effect of SMAD3 inhibition by siSMAD3 in primary myometrial cells was a significant increase in the expression of proinflammatory cytokines IL-1*α*, IL-6, IL-8, and MCP-1 and the release of IL-6, IL-8, and MCP-1 in the presence of IL-1*β* and poly(I : C). SMAD3 knockdown also increased adhesion molecule ICAM-1 expression and secretion but not VCAM-1 in myometrial cells. Furthermore, COX-2 and FP mRNA expression and PGF_2*α*_ release was also significantly increased following SMAD3 inhibition.

Studies in nongestational tissues have shown that inflammation is associated with reduced SMAD3 function. In this study, activated SMAD3 (p-SMAD3) expression was decreased in the myometrium after term labour and delivery. This decrease in the abundance of the active form of SMAD3 in the myometrium with labour suggests that SMAD3 may play a role in human labour. Human labour is considered to be an inflammatory process, with studies showing a significant increase in IL-1*β*, IL-6, and IL-8 mRNA expression in the myometrium of labouring women [43, 44]. This increase in proinflammatory cytokines in the myometrium induces uterine contractions [45]. Indeed, in a nonhuman primate model IL-1*β* induces preterm labour [7]. Pathological insults such as viral infection have also been shown to induce inflammation in uterine tissue. For example, influenza virus infection of human uterine cervical fibroblasts increases expression of IL-1*β*, IL-6, and TNF *in vitro* [46]. We have previously demonstrated that the viral dsRNA synthetic mimetic poly(I : C) upregulates the secretion of proinflammatory cytokines and prolabour mediators in the human myometrium [37]. In addition, *in vivo* studies have reported that administration of poly(I : C) induces preterm delivery within 24 h in pregnant mice [6]. Thus, we next sought to assess the expression of SMAD3 to inflammation and pathological insults in the human myometrium.

IL-1*β* has been shown to suppress TGF-*β*-induced anti-inflammatory signalling [47]. With respect to labour and delivery, TGF-*β* inhibits prostaglandin production in amnion cells [48] and rescues IL-1*α*- and TNF-*β*-induced preterm birth in rabbits [49]. In the present study, IL-1*β*, TNF, and poly(I : C) significantly downregulated SMAD3 expression in the myometrium, indicating a mechanism by which SMAD3 may be decreased during labour. We also confirmed that TGF-*β* is able to phosphorylate and activate Smad3 to induce transcription; however, IL-1*β*, TNF, and poly(I : C) had no effect upon TGF-*β* stimulation (data not shown). Furthermore, there was no effect of TGF-*β* on basal or IL1B-, TNF-, fsl-1, or poly(I : C)-induced markers of inflammation in human primary myometrial cells (data not shown) suggesting that the effects are independent of TGF-*β*. Independently from TGF-*β* signalling, there are numerous signalling pathways and mediators that influence the transcriptional activity of SMAD3. Studies have shown that SMAD3 activity may be regulated by the following: (i) phosphorylation by mitogen-activated protein kinases (MAPKs); (ii) interactions with transcriptional cofactors (i.e., coactivators or corepressors); or (iii) negative feedback loops by I-SMADS (i.e., SMAD7). Oncogenic hyperactive Ras has been shown to inhibit TGF-*β*-induced nuclear accumulation of SMAD3 and SMAD-dependent transcription [50]. Activation of the ERK/MAPK pathway by Ras induces phosphorylation of the linker region of SMAD3 which is distinct from the TGF-*β* receptor phosphorylation sites. Linker phosphorylation inhibits nuclear translocation of TGF-*β*-activated SMAD3 [50]. Transcription factors of the AP-1 family may also interact with the R-SMAD/SMAD4 complex in the nucleus. c-Jun and JunB, which are components of the AP-1 complex and are downstream substrates of JNK, can interrupt SMAD3-driven transcription by forming Jun/SMAD3 complexes to prevent SMAD3 from binding to DNA [51]. In addition, c-Jun can bind to the transcriptional coactivators CBP/p300 to prevent SMAD3-mediated transcription by sequestering CBP/p300 away from R-SMAD/SMAD4 complexes [51].

We next performed loss-of-function studies to assess the role of SMAD3 in the production of proinflammatory and prolabour mediators induced by IL-1*β* and poly(I : C). In human pregnancy and parturition, cytokines and chemokines play an important role. The onset of labour is associated with a large influx of leukocytes, such as neutrophils and macrophages, in the myometrium. This facilitates the production of proinflammatory mediators while decreasing the production of anti-inflammatory cytokines [43, 52, 53]. This shift to increasing proinflammatory cytokines and chemokines induces the production of prostaglandins and matrix metalloproteases (MMPs) which in turn activates cervical ripening and uterine contractions, resulting in labour and delivery. Studies in nongestational tissue demonstrate that SMAD3 exerts protective effects against inflammation. For example, homozygous SMAD3 knockout mice had higher mortality in response to LPS-induced endotoxemia compared to wild-type mice [17]. In this study, knockdown of SMAD3 using siRNA shows that SMAD3 regulates the inflammatory response to proinflammatory cytokines or TLR3 activation in the human myometrium. Specifically, IL-1*β* or poly(I : C)-stimulated expression of the proinflammatory cytokines IL-1*α* and IL-6 and the chemokines IL-8 and MCP-1 was further increased in siSMAD3-transfected myometrial cells. Collectively, these studies report an important role for SMAD3 in regulating the inflammatory response in response to sterile inflammatory insults.

Cell adhesion molecules are well described to be essential for the recruitment and chemotaxis of infiltrating maternal leukocytes to the myometrium, in order to augment inflammation and potentiate uterine contractions that subsequently lead to human labour and delivery [54]. As a result of proteolytic cleavage at the cell surface, soluble forms of ICAM-1 and VCAM-1 are detectable in the systemic circulation. Women who deliver preterm are associated with elevated levels of circulating sICAM-1 and sVCAM-1 compared to women who deliver at term [55]. In nongestational tissue, the TGF-*β*/SMAD pathway has been shown to downregulate vascular inflammation and atherogenesis [16]. In gestational tissue, a recent study reported that inhibition of SMAD3 significantly augmented ICAM-1 and VCAM-1 expression using human umbilical vein endothelial cells (HUVECs) [15]. In corroboration, we found that expression and secretion of sICAM-1 and sVCAM-1 in human myometrial cells were significantly elevated with IL-1*β* and poly(I : C) treatment. This increase in sICAM-1 was further elevated with the loss of SMAD3. There was, however, no effect on sVCAM-1, which might suggest that other mechanisms independent of SMAD3 are involved in regulating VCAM-1 expression in myometrial cells.

The COX-2/prostaglandin pathway plays an important role in labour initiation and progression by stimulating myometrial and cervical ripening [56]. COX-2 is responsible for prostaglandin synthesis and is upregulated in gestational tissues during labour [57] and inflammation [58]. FP is a receptor for the prostaglandin PGF_2*α*_, and when activated, modulates calcium influx into the myometrium to initiate uterine contractions. Term labour is associated with elevated FP receptor mRNA expression in the human myometrium [59]. There is conflicting evidence on the role of the TGF-*β*/Smad pathway in regulating COX-2 expression. For example, in human granulosa cells, TGF-*β*1 induces COX-2 expression and PGE_2_ production via SMAD2 and SMAD3 signalling [60]. In contrast, other studies have shown that the TGF-*β*/Smad pathway suppresses COX-2 expression. For example, homozygous SMAD3 knockout mice are associated with the overexpression of COX-2 [61], and in A549 human lung cancer cells, TGF-*β* downregulated the COX-2 expression via mRNA destabilisation through SMAD3 [62]. In the present study, the loss of SMAD3 significantly amplified IL-1*β* and poly(I : C)-stimulated COX-2 and FP mRNA expression and PGF_2*α*_ secretion. The discrepancy in the literature on the role of SMAD3 in regulating the COX-2/prostaglandin pathway might be due to differing regulatory mechanisms employed by different tissue and cell types. Nevertheless, to our knowledge, this is the first study to show that SMAD3 suppresses the COX-2/prostaglandin pathway in primary myometrial cells.

The limitations of this study are that functional studies were performed only by gene loss-of-function. While there was no effect of TGF-*β* stimulation on prolabour mediators in myometrial cells (data not shown), Smad3 overexpression should lead to accordant observations. Furthermore, due to inadequate samples, we cannot establish if preterm labour is also associated with decreased SMAD3 activity. The relationship between SMAD3 and other SMADs is also worth examining, as I-SMADs have been shown to regulate SMAD3 activity [63–65]. For example, SMAD7 acts as an inhibitor of TGF-*β* signalling by preventing SMAD3 phosphorylation by the TGF-*β* receptor. SMAD7 also interferes with R-SMAD4 complex formation by competing with SMAD4 to interact with R-SMADs [65, 66]. Indeed, we have reported that the blockade of SMAD7 decreases the synthesis of proinflammatory and prolabour mediators in the human myometrium [32]. Further studies are required to determine the relationship between SMAD3 and SMAD7 in regulating proinflammatory and prolabour mediators.

In conclusion, this study demonstrates for the first time that SMAD3 is reduced with spontaneous term labour. SMAD3 deficiency in myometrial cells results in an exacerbation of IL-1*β* and poly(I : C)-induced proinflammatory and prolabour mediators, indicating that SMAD3 is a negative regulator of the inflammatory response in the myometrium. This is an important finding given the strong links between inflammation and preterm birth [4, 5]. Further studies are required for improving our understanding of the role of SMAD3 in parturition which may lead to more effective interventions against spontaneous preterm birth.

## Data Availability

The data used to support the findings of this study are available from the corresponding author upon request.

## Supplementary Materials

Supplementary Materials The supplementary material is a figure showing the efficacy of siRNA SMAD3 knockdown in primary myometrial cells on SMAD3 mRNA and protein expression.
