# Supplementary material for: Identification of SMAD3 as a Novel Mediator of Inflammation in Human Myometrium In Vitro
Source: Mediators Inflamm. 2018 Sep 27;2018:3140420. doi: 10.1155/2018/3140420 (PMC6180979; doi:10.1155/2018/3140420)
Supplement: Supplementary Materials — The supplementary material is a figure showing the efficacy of siRNA SMAD3 knockdown in primary myometrial cells on SMAD3 mRNA and protein expression. [file 3140420.f1.docx]

**Supplementary Figure 1. Efficacy of siSMAD3 knockdown**

Human primary myometrial cells were transfected with or without 50 nM siSMAD3 or siCONT for 48 h (n=5 patients). **(A)** SMAD3 mRNA expression was analysed by qRT-PCR and the fold change was calculated relative to the siCONT transfected cells. Data is displayed as mean ± SEM. **P*<0.05 vs. siCONT (paired sample analysis). **(B)** SMAD3 protein expression was analysed by Western blot and the fold change was calculated to the siCONT transfected cells. Data is displayed as mean ± SEM. **P*<0.05 vs. siCONT transfected cells (paired sample analysis). Representative Western blot from 1 patient is also shown. **(C)** Cell viability was assessed using a MTT assay and the fold change was calculated relative to the siCONT transfected cells. Data is displayed as mean ± SEM. **P*<0.05 vs. siCONT (paired sample analysis).
